# Machine learning identification of cuproptosis and necroptosis-associated molecular subtypes to aid in prognosis assessment and immunotherapy response prediction in low-grade glioma

**DOI:** 10.3389/fgene.2022.951239

**Published:** 2022-09-12

**Authors:** Ye Miao, Jifeng Liu, Xishu Liu, Qihang Yuan, Hanshuo Li, Yunshu Zhang, Yibo Zhan, Xiaoshi Feng

**Affiliations:** ^1^ Department of Neurosurgery, The First Affiliated Hospital of Jinzhou Medical University, Jinzhou, Liaoning, China; ^2^ Department of Surgery, First Affiliated Hospital of Dalian Medical University, Dalian, Liaoning, China; ^3^ Department of Thoracic Surgery, First Affiliated Hospital of Dalian Medical University, Dalian, Liaoning, China; ^4^ Department of Endocrinology, The First Affiliated Hospital of Jinzhou Medical University, Jinzhou, Liaoning, China

**Keywords:** cuproptosis, necroptosis, prognostic signature, immunotherapy, low-grade glioma

## Abstract

Both cuproptosis and necroptosis are typical cell death processes that serve essential regulatory roles in the onset and progression of malignancies, including low-grade glioma (LGG). Nonetheless, there remains a paucity of research on cuproptosis and necroptosis-related gene (CNRG) prognostic signature in patients with LGG. We acquired patient data from The Cancer Genome Atlas (TCGA) and Gene Expression Omnibus (GEO) and captured CNRGs from the well-recognized literature. Firstly, we comprehensively summarized the pan-cancer landscape of CNRGs from the perspective of expression traits, prognostic values, mutation profiles, and pathway regulation. Then, we devised a technique for predicting the clinical efficacy of immunotherapy for LGG patients. Non-negative matrix factorization (NMF) defined by CNRGs with prognostic values was performed to generate molecular subtypes (i.e., C1 and C2). C1 subtype is characterized by poor prognosis in terms of disease-specific survival (DSS), progression-free survival (PFS), and overall survival (OS), more patients with G3 and tumour recurrence, high abundance of immunocyte infiltration, high expression of immune checkpoints, and poor response to immunotherapy. LASSO-SVM-random Forest analysis was performed to aid in developing a novel and robust CNRG-based prognostic signature. LGG patients in the TCGA and GEO databases were categorized into the training and test cohorts, respectively. A five-gene signature, including SQSTM1, ZBP1, PLK1, CFLAR, and FADD, for predicting OS of LGG patients was constructed and its predictive reliability was confirmed in both training and test cohorts. In both the training and the test datasets (cohorts), higher risk scores were linked to a lower OS rate. The time-dependent ROC curve proved that the risk score had outstanding prediction efficiency for LGG patients in the training and test cohorts. Univariate and multivariate Cox regression analyses showed the CNRG-based prognostic signature independently functioned as a risk factor for OS in LGG patients. Furthermore, we developed a highly reliable nomogram to facilitate the clinical practice of the CNRG-based prognostic signature (AUC > 0.9). Collectively, our results gave a promising understanding of cuproptosis and necroptosis in LGG, as well as a tailored prediction tool for prognosis and immunotherapeutic responses in patients.

## Introduction

Gliomas are cancerous forms that develop in the central nervous system (CNS). The World Health Organization (WHO) has categorized gliomas into 4 grades, where gliomas classified as grades II and III are considered low-grade gliomas (LGG) (Louis et al., 2016). In addition to a long history of ionizing radiation, the risk factors for LGG are not fully understood (Salvati et al., 1991). LGG proliferates and progresses in a variety of ways, and patients’ quality of life and survival rate is poor (Cancer Genome Atlas Research et al., 2015; Youssef and Miller, 2020). The median overall survival (OS) durations for individuals with LGG were 78.1 and 37.6 months (Jiang et al., 2016). Although significant progress has been made in developing innovative cancer therapies, the prognosis for LGG patients remains dismal. Immunotherapy has been largely regarded as a viable treatment for a variety of cancers. As a result, the development and validation of new prognostic markers to better predict clinical outcomes and immunotherapy in LGG patients remain an urgent need.

Necroptosis is a newly established kind of cell death that is triggered by Receptor Interacting Protein Kinase1/3 and carried out by Mixed Lineage Kinase Domain Like Pseudokinase (Declercq et al., 2009; Marshall and Baines, 2014). Necroptosis is both a friend and a foe in tumour growth, as earlier research has shown (Philipp et al., 2016). On the one hand, if cancer cells can evade apoptosis, it may serve as a supplementary kind of programmed cell death. Necroptosis, on the other hand, can activate the inflammatory response, which can lead to tumour development (Gong et al., 2019). In addition, necroptosis, one of the immunogenic cell deaths, performs a critical function in the immune microenvironment (Sprooten et al., 2020). In the context of the rise of immune checkpoint therapy, changes in the immune microenvironment caused by necrosis are also important (Li et al., 2019a). Tsvetkov et al. (2022) have discovered that intracellular copper causes “cuproptosis,” a new type of controlled cell death distinct from cell death linked to oxidative stress (such as necroptosis, ferroptosis, and apoptosis). Copper binds directly to lipoylated components of the tricarboxylic acid (TCA) cycle, causing cuproptosis. This leads to the aggregation of lipoylated proteins and, as a consequence of that, the loss of iron-sulfur cluster proteins, ultimately leading to proteotoxic stress and cell death (Tsvetkov et al., 2022). To our knowledge, the prognostic performances of necroptosis and cuproptosis in LGG remain unclear. Necroptosis and cuproptosis have not yet been looked at in depth to see how they affect LGG as a whole. Therefore, it is vital to further investigate the connection between cuproptosis and necroptosis-related genes (CNRGs) and LGG.

In this research, we used the gene expression levels and clinical data from the Chinese Glioma Genome Atlas (CGGA) and The Cancer Genome Atlas (TCGA) databases to examine CNRGs. Non-negative matrix factorization (NMF) defined by CNRGs with prognostic values was performed to separate LGG patients into entirely different subgroups with considerably different prognoses, clinical features, immunological microenvironments, and immunotherapy responses. LASSO-SVM-random Forest analysis was performed to aid in developing a novel and robust CNRG-based prognostic signature. According to the TCGA and CGGA cohorts, we created and validated a risk-score system for LGG with the optimal prognostic performance. Furthermore, the mechanism of action and pathways of cuproptosis and necroptosis-related genes were further analysed by immune checkpoint gene expression, immune subtype identification, immune cell infiltration, tumour mutation profile, tumour stemness indices, and immunotherapy response analysis. Based on the immunohistochemistry findings, we verified the differential expression of associated genes encoding proteins in LGG in the model. Our results gave a promising understanding of cuproptosis and necroptosis in LGG, as well as a tailored prediction tool for prognosis and immunotherapeutic responses in patients.

## Materials and methods

### Acquisition of datasets and cuproptosis and necroptosis-related genes

In all, this study included 1154 LGG samples. The TCGA (https://portal.gdc.cancer.gov/) and CGGA (http://www.cgga.org.cn/) databases were searched to acquire RNA-seq and clinical data. In particular, the TCGA-LGG dataset (529 LGG samples, 56,753 genes) was designated as the training cohort, whereas the CGGA dataset (625 samples, 23,271 genes) was the validation cohort. All data from the TCGA and CGGA databases were transformed into log2 (x + 1) form for subsequent analyses. After that, the data on gene expression profiles from various databases were batch-normalized utilising the “Surrogate Variable Analysis (sva)” program that is included in R (Li et al., 2021). After intersecting all genes from the two datasets, 17,818 genes were defined as common genes. All data were provided in an open-access format, thus ethics committee permission was not necessary.

The 74 necroptosis-related genes and 13 cuproptosis-related genes were reported by previous studies (Tsvetkov et al., 2022; Xin et al., 2022). After eliminating two genes (CXCL8 and OTULIN) without expression levels in the CGGA and TCGA datasets, we integrated the expression profiles of the remaining 85 CNRGs with those of the TCGA and CGGA cohorts for further analysis (TCGA: Supplementary Table S1, CGGA: Supplementary Table S2).

### Pan-cancer analysis

Changes in the gene expression patterns of CNRGs [|log2(FC)| > 1, FDR 0.05] were investigated using differential expression analysis between tumours and neighbouring normal tissues for each cancer type. The CNRGs’ survival landscape was derived from TCGA’s analysis of the link between gene expression and patient survival. The criteria for a protective gene were set as a hazard ratio (HR) < 1 and *p* < 0.05, whereas the criteria for a risk gene were HR > 1 and *p* < 0.05.

To identify significantly altered regions of amplification or deletion across patient groups, GISTIC2.0 was utilised to analyse copy number variation (CNV) data of 11495 samples obtained from TCGA database. The percentage of genes that were heterozygous or homozygous for CNVs was shown using a CNV pie plot for each tumour type. This pie chart illustrates the distribution of CNV types inside a single malignancy, with each hue denoting a distinct CNV type.

Single nucleotide variation (SNV) data for 10,234 samples across 33 types of cancers were also acquired from TCGA database. Within the scope of this study, seven distinct forms of mutation were considered: In_Frame_Ins, In_Frame_Del, Frame_Shift_Del, Frame_Shift_Ins, Splice_Site, Nonsense_Mutation, and Missense_Mutation. Mutation frequencies in pan-cancer were summarised using a percentage heatmap. Finally, gene set enrichment analysis (GSEA) was utilised to examine the cellular signatures characteristic of each malignancy.

### Non-negative matrix factorization clustering determination of cuproptosis and necroptosis-related gene modification subtypes

Univariate cox regression analysis was conducted to screen the genes with prognostic values in both the derivation and validation cohorts, reducing the dimensionality of NMF clustering. Only genes involved in prognosis that are strongly associated with cuproptosis and necroptosis were kept as clustering factors for NMF.

To examine the link between CNRG expression and clinical characteristics in LGG, we grouped TCGA LGG samples into two distinct groups (clusters 1 and 2) utilising NMF. NMF was designed to uncover possible features in gene expression patterns by resolving the initial matrix into two nonnegative matrices. The process of deposition was repeated several times, and the results of each iteration were aggregated to get a consensus cluster of LGG samples. The silhouette, dispersion, and cophenetic coefficients were utilised to ascertain the optimum number of subtypes. The range of values for the number of clusters, k, was selected to be between 2 and 10, and the “NMF” package was employed to establish the average contour width of the common member matrix.

### Discrepancies in the clinical characteristics, tumour immune microenvironment and immunotherapy response between distinct cuproptosis and necroptosis-related gene-based clusters

The prognostic efficacy of clusters was assessed using Kaplan-Meier analyses (Du et al., 2021), with the progression-free interval (PFI), disease-specific survival (DSS), and overall survival (OS), as endpoints. We also intensively explored the discrepancies in the clinical information between distinct CNRG-based clusters. The immune and stroma scores were derived by Estimation of Stromal and Immune cells in Malignant Tumour tissues using Expression data (ESTIMATE) analysis utilising the “estimate” R package (Guo and Jing, 2021). The algorithm also allowed for the determination of the level of tumour purity. At the same time, to ascertain the abundance of immune cells that had been infiltrated into each sample, the CIBERSORT algorithm was used. Following this, the “Wilcox.test” function in R was adopted to explore the disparity between infiltration levels of immune cells and typically immune checkpoint genes (ICGs, Supplementary Table S3). The tumour immune dysfunction and exclusion (TIDE, http://tide.dfci.harvard.edu/) algorithm was employed to forecast probable responses to ICI treatment. TIDE is a gene expression biological marker utilised to predict the patients’ responsiveness to immune checkpoint inhibition. A low exclusion score indicates a low probability of immune evasion; hence, these individuals may display a stronger immune treatment response.

### Machine learning-based development and validation of the optimal cuproptosis and necroptosis-related gene risk signature

An integrated analysis of two algorithms was used to choose the putative prognostic CNRGs. These algorithms were the LASSO algorithm with penalty parameter tuning performed by 10-fold cross-validation and the SVM-RFE algorithm screening for lambda with the minimized classification error to obtain the variable. An additional filtering method known as the Random Survival Forests-Variable Hunting (RSFVH) algorithm was implemented to filter the genes. Thereafter, the following is a description of how a risk score model was created utilising prognostic genes: $\overset{n}{\underset{k=1}{\sum }}expk*\beta k$ (n denotes the number of genes chosen using RSFVH, “expk” denotes the gene expression value, and “βk” denotes the coefficient of genes acquired from the Cox regression analysis. The log-rank *p*-values were employed in the Kaplan-Meier (KM) analysis to search for the optimal gene combination or the final signature.

After determining the suitable threshold value for the risk score, patients within TCGA cohort were categorized into low- and high-risk groups utilising the “survival” and “survminer” software packages. Following the calculation algorithm and median risk score supplied in TCGA cohort, we derived each LGG sample’s risk score within the CGGA cohort and subsequently classified these samples into low- and high-risk groups. The KM technique was applied to generate survival curves illustrating the disparities in expected survival time and probability across the high- and low-risk patients in both CGGA and TCGA datasets. With the aid of the “timeROC” package in R (Chen and Li, 2022), the ROC curves were plotted, and the area under the curves (AUC) for 1-, 3-, 5-, and 7-year OS were computed for both the CGGA and TCGA cohorts. To additionally test the viability of the risk score-based predictive model in patients with LGG in both the CGGA and TCGA datasets, the principal component analysis (PCA) and the t-distributed stochastic neighbour embedding (t-SNE) analyses were done. Moreover, the predictive capacity of our CNRG prognostic signature was subjected to a comparison with other three well-recognized prognostic signatures (hypoxia-related prognostic signature constructed by Lin et al. (2020), an immune-related prognostic signature constructed by Zhang et al. (2020), an RNA methylation-related prognostic signature designed by Zheng et al. (2021).

The single sample gene set enrichment analysis (ssGSEA) was utilised to estimate scores premised on five model genes in each sample of each tumour. This was done to determine the differential function that our signature performs in the pathways that are altered by human multiple malignancies. As per the transcriptomes of two different tumour groups, one with the highest and another with the lowest 30% of scores, GSEA was applied to explore the discrepancy in cuproptosis, necroptosis, and classical pathway activities.

### Discrepancies in the clinical traits, immune traits, and tumour stem traits in low- and high-risk low-grade glioma individuals

Fisher tests were used to illustrate the distributional variations in histological type, gender, survival status, age, cancer status, and grade between low- and high-risk groups to analyse the association between the CNRG prognostic signature and clinicopathological features. The immunological differences (variations) between low- and high-subgroups were investigated. Estimate algorithm (estimate of cancerous and immune cells present in malignant tumour organization utilising expression profiling) is employed to examine the percentage of immune-matrix components in tumour immune microenvironment (TIME), encompassing ESTIMATE Score (total score taking into account both immunity and matrix), Immune Score (indicating the degree to which immune cells have been infiltrated), and Stromal Score (an indication of the existence of matrix). When the score is higher, it implies that there is a larger concentration of the TIME component. TIMER database was used to calculate the levels of immune-infiltrating cells through multiple immunological algorithms, such as XCELL, CIBERSORT, MCPCOUNTER, CIBERSORT-ABS, and TIMER. ICGs play pivotal roles in regulating the function of immune cells, thus, we further intensively analysed the discrepancies in the expression of ICGs between the low- and high-risk subpopulations. Thorsson et al. (2018) summarised six immune subtypes (C1-C6) for pan-cancer samples derived from the TCGA database. In 2018, Malta et al. (2018) evaluated the DNA stemness scores (DNAss) and RNA stemness scores (RNAss) with the help of the one-class logistic regression (OCLR) machine learning method. Therefore, we also compared the discrepancy in immune subtypes, DNAss, and RNAss between high- and low-risk populations.

### Discrepancies in the tumour mutation traits in low- and high-risk groupings

The relevant data on the somatic alteration data of the TCGA-LGG cohort was taken from TCGA dataset. The waterfall plot was utilised to demonstrate the relative mutation profiling of the low- and high-risk groups and was generated with the “maftools” R package. Thereafter, the Wilcox test was applied to compare the two groups in regard to the differences in the mutation frequencies. In addition, TMB was computed for each patient, and a Spearman correlation analysis with estimated *p*-values was utilised to determine the specific association between CNRGs and TMB. Notably, we also examined the survival value of TMB in terms of OS in the LGG population.

### Independent prognostic analysis of the cuproptosis and necroptosis-related gene signature and nomogram development

Thereafter, we checked whether clinical pathological parameters, such as age, histological grade, and cancer status, had an impact on the predictive capacity of the CNRG signature. To determine which factors influence a patient’s prognosis independently, univariate and multivariate Cox regression analyses were undertaken on TCGA cohort. Variables were considered to be independent prognostic factors if they had a *p* value < 0.05. With rms R package (Hu et al. 2022), the nomogram was set up premised on the above clinicopathological factors, CNRGs, and our signature. The objective of the nomogram was to examine the predictive significance of the risk score obtained for 1-, 3-, 5-, and 7-year OS rates.

### Immunohistochemistry and immunofluorescence of the model genes in low-grade glioma

The Human Protein Atlas (HPA) is a database that contains protein expression patterns premised on immunohistochemistry (IHC) that were collected from cell lines, normal tissues, and cancer tissues (Ponten et al., 2008). This database was used to acquire protein expression IHC pictures of model genes in clinical samples from LGG patients for the current investigation. Similarly, the HPA database was also employed to demonstrate the cellular localization (SQSTM1, CFLAR, and FADD) through immunofluorescence.

## Results

### Expression traits, prognostic values, copy number variation, Single nucleotide variation and cancer signalling of the cuproptosis and necroptosis-related genes in cancers according to the cancer genome atlas

**FIGURE 1:**
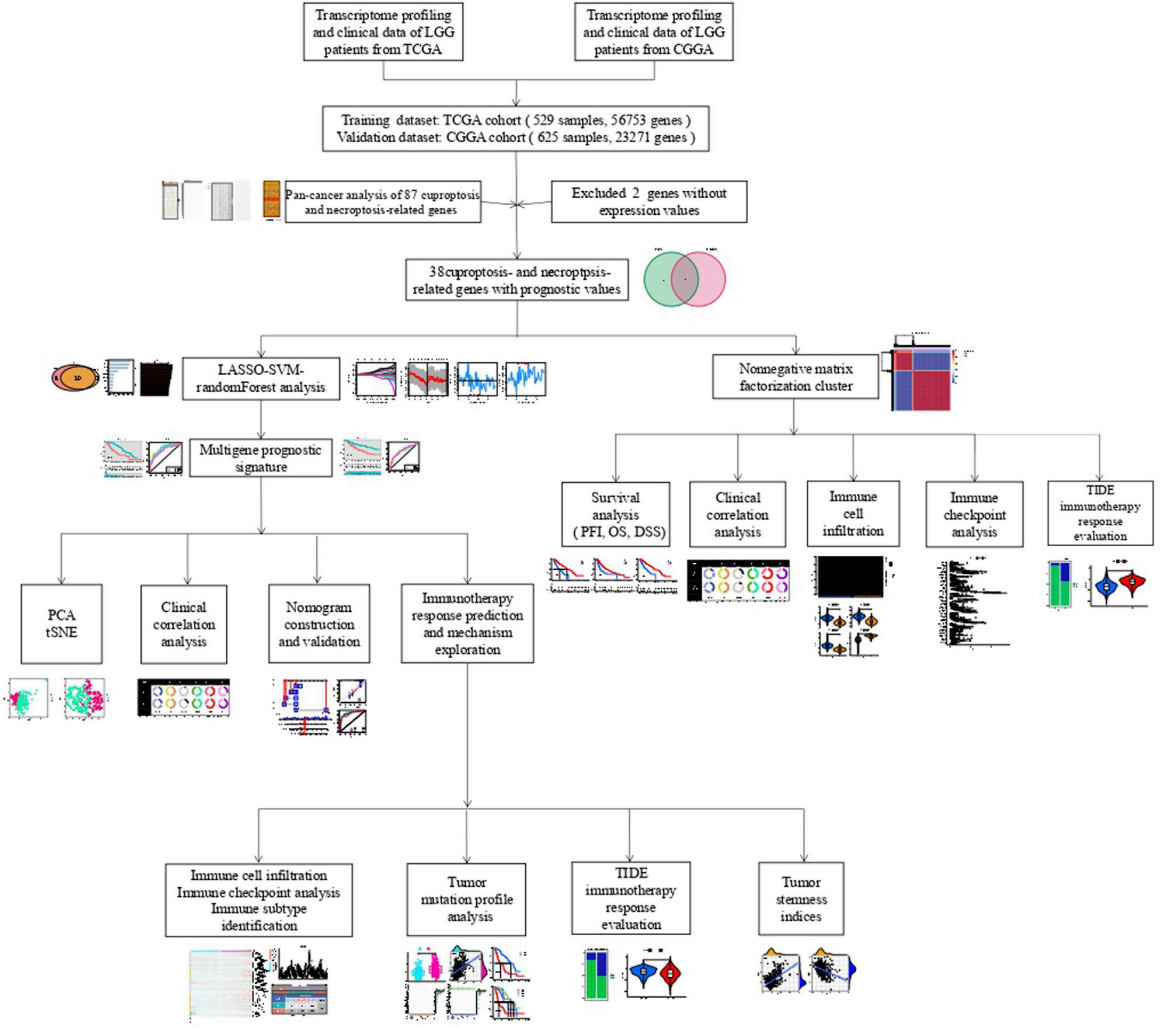
The flow chart of this investigation.

**FIGURE 2:**
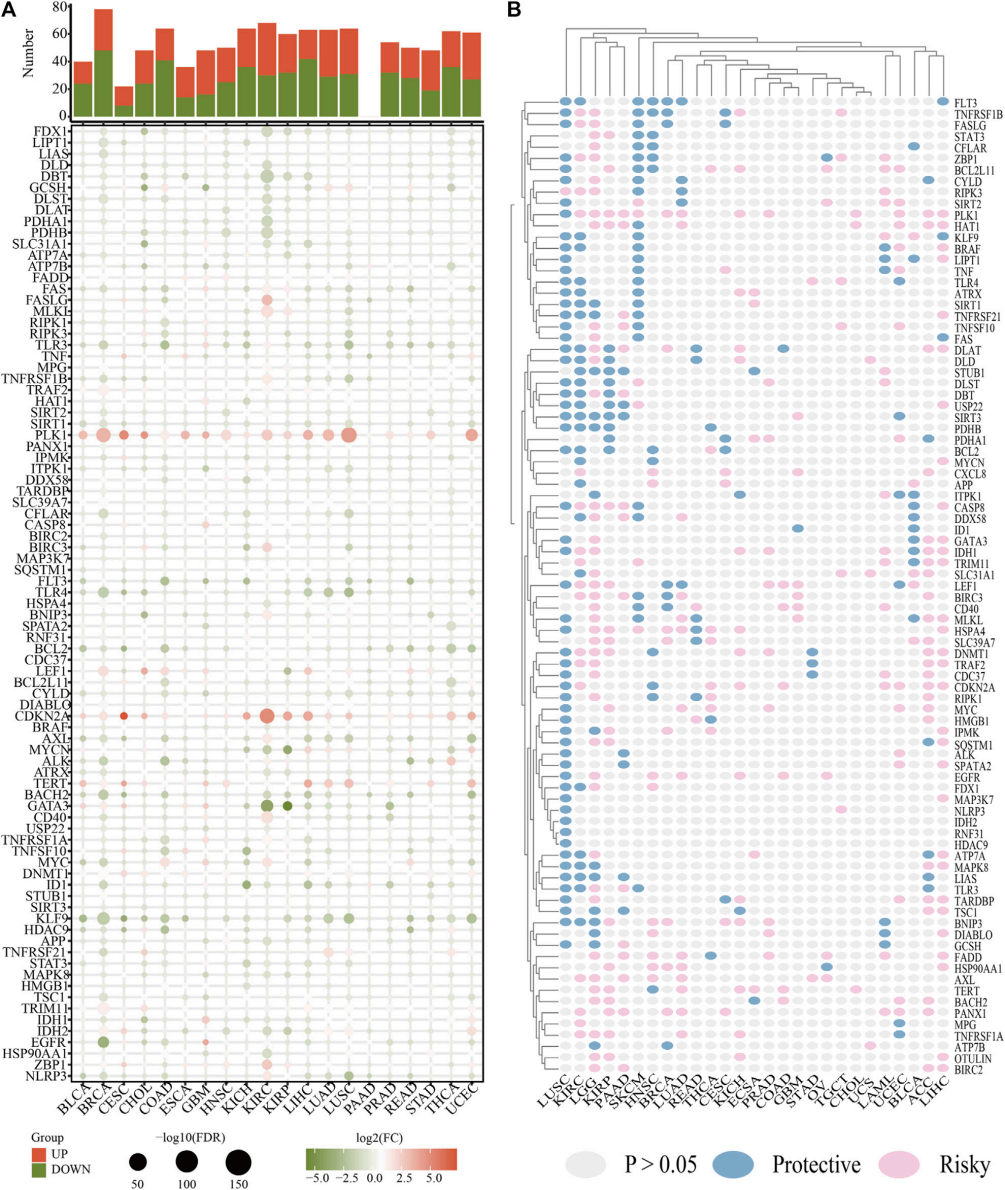
The roles of CNRGs in cancer. **(A)** The heatmap depicts the fold change and FDR of CNRGs in each tumour, whereas the histogram (top panel) shows the number of significantly differentially expressed genes. **(B)** Heatmap showed the survival landscape of CNRGs.

Figure 1 depicts the flow chart for this research. Since the correlation between cuproptosis and necroptosis is unclear, we first performed a co-expression analysis of CNRGs. The findings illustrated that the correlation between the expression levels of CNRGs was significant in both TCGA and CGGA cohorts (Supplementary Figure S1). 85 well-recognized CNRGs with complete expression values both in the CGGA and TCGA cohorts were included in the following analysis. The role of cuproptosis and necroptosis in tumour progression has not been clarified, and pan-cancer characterization of necroptosis and cuproptosis-related genes are not well summarized. Thus, intensive exploration of the contributions of these genes in diverse human malignancies from the perspective of expression traits, prognostic values, cancer signalling, CNV and SNV would therefore be highly warranted. We discovered that in cancerous tissues, the expression of a majority of genes differed from those in normal tissues (Figure 2A). The expression patterns of PLK1 and CDKN2A were considerably up-modulated in most tumour types, while KLF9 was the opposite. After that, we constructed a survival landscape of these genes based on the link between the gene expression levels and the patient survival rates recorded in TCGA (Figure 2B). HR < 1 and *p* < 0.05 indicate a protective gene, whereas HR > 1 and *p* < 0.05 indicated a risk gene. We found that most of the genes in LUSC, KIRC, LGG, and LIHC were associated with patient prognosis. Most of the protective genes were found in LUSC and KIRC, while most of the risk genes were in LGG and LIHC. Meanwhile, the SNV and CNV alterations of cuproptosis and necroptosis-related genes in pan-cancer including LGG were obvious (Supplementary Figures S2, S3). BRAF, ATRX, IDH1, and CDKN2A showed significant SNV alterations in most tumour types. GEGFR, CD40, SPATA2, ZBP1, ID1, and MYC showed a significant CNV amplification in most tumour types; however, TLR3, PDHB, FAS, and MAP3K showed a significant CNV deletion in most tumour types. Considering the unclear role of necroptosis and cuproptosis in LGG and the fact that most CNRGs are linked to unfavourable LGG patients’ prognoses, we focused on the relationship between CNRGs and LGG. The relationship between cancer signalling and CNRGs was also investigated, with the findings revealing that 50 hallmarks were frequently strongly linked to CNRGs (Supplementary Figure S4). For example, interferon *γ* response, interferon *α* response, IL2 STAT5 signalling, inflammatory response, and allograft rejection were enriched in each cancer, which indicated that cuproptosis and necroptosis were positively related to these oncogenic pathways. Interestingly, most oncogenic pathways were significantly enriched in LGG.

### Data acquisition and processing

**FIGURE 3:**
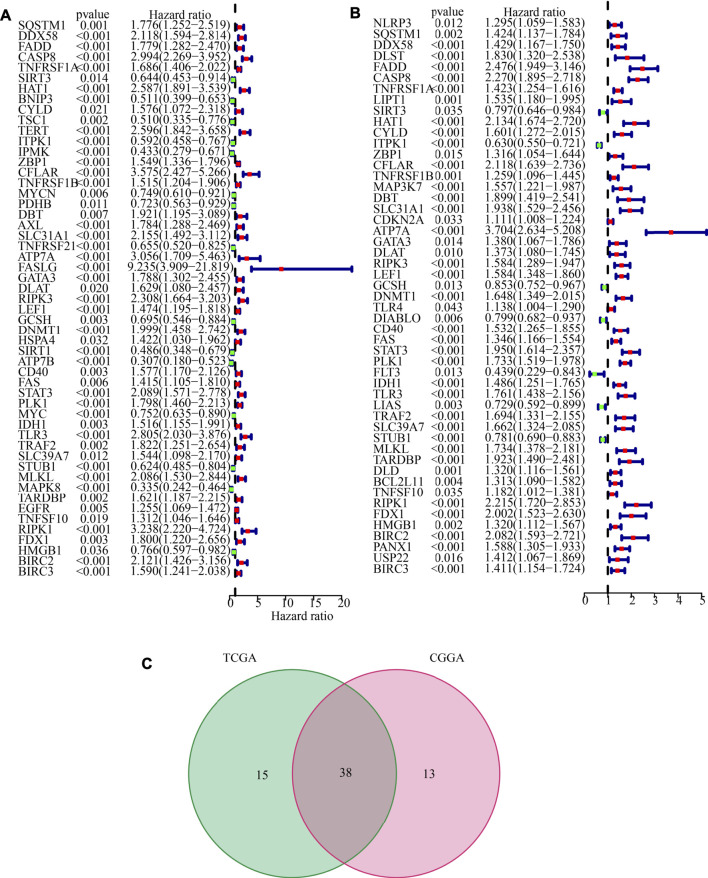
Identification of prognostic cuproptosis and necroptosis-related genes (CNRGs). **(A)** 53 CNRGs with prognostic values in TCGA dataset. **(B)** 51 CNRGs with prognostic values in the CGGA dataset. **(C)** Venn diagram to identify 38 FPRGs with prognostic values in LGG.

The LGG RNA-Seq data from TCGA constituted the training set, whereas the LGG RNA-Seq data from CGGA constituted the validation set. 53 CNRGs in the TCGA cohort and 51 CNRGs in the CGGA cohort were chosen using univariate cox regression analysis and false discovery rate adjustment (Figures 3A,B). After obtaining an intersection between the 51 prognosis CNRGs and the 53 prognostic CNRGs, 38 CNRGs having prognostic values were found (Figure 3C).

### Non-negative matrix factorization clustering identification of molecular typing based on the shared cuproptosis and necroptosis-related genes with prognostic values

**FIGURE 4:**
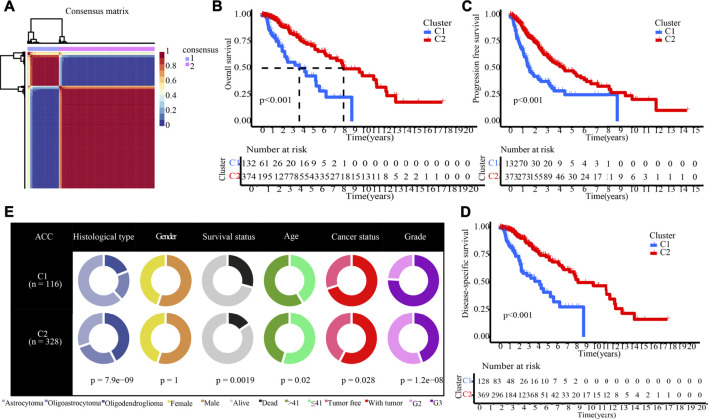
NMF clustering yields two molecular subtypes with significantly different prognoses and clinical characteristics. **(A)** The optimal clustering number of 2. **(B–D)** Kaplan-Meier analyses (OS, PFI, and DSS) as regards two molecular subtypes. **(E)** Pie charts illustrating the Chi-squared test of clinical and pathologic features between two molecular subtypes.

**FIGURE 5:**
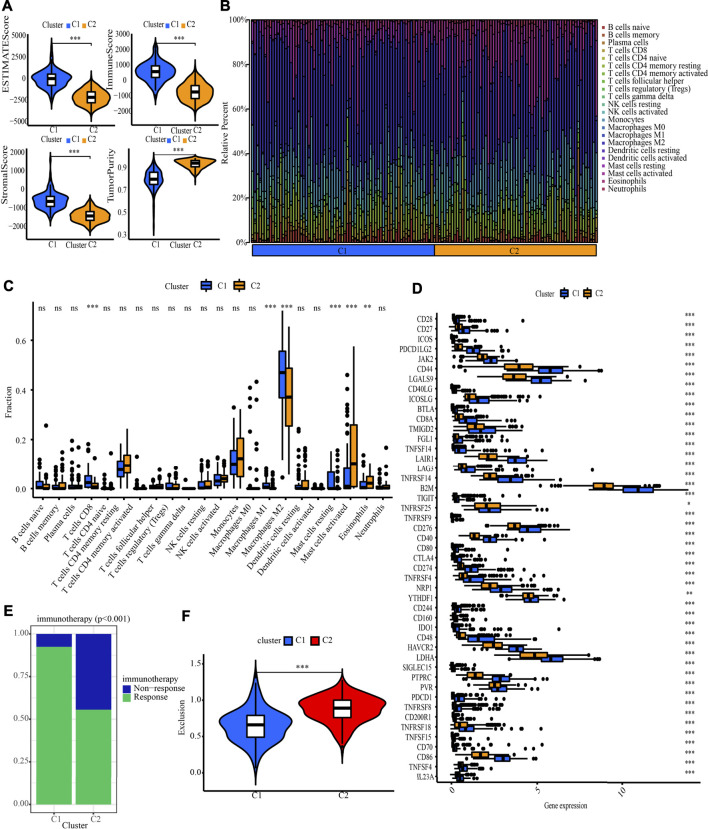
Systematic analysis of TME scores, immune cell infiltration and immunotherapy response prediction in two molecular subtypes. **(A)** Comparison of TME components. **(B)** The proportion of 22 subsets of tumour-infiltrating immune cells in distinct subtypes. **(C)** Discrepancy analysis of tumour infiltrating immune cells in distinct subtypes **(D)** Differential expression analysis of 47 immune checkpoints genes between two molecular subtypes. **(E)** The discrepancy of immunotherapy response in two subgroups. **(F)** Immunotherapy response prediction in two subgroups.

The NMF method selects the appropriate clustering number of 2 for the data, as per cophenetic, dispersion, and silhouette coefficients (Supplementary Figures S5, S6, Figure 4A). Through KM analyses, it was found that the samples in cluster 2 (C2) have better OS, PFI, and DSS (Figures 4B–D). The examination of the compositional differences in clinical features (Figure 4E), indicates that there are more astrocytoma samples in C1 and more oligodendroglioma samples in C2 (*p* = 7.9e-09). Furthermore, when compared to C2, C1 had a higher proportion of dead patients (*p* = 0.0019), elderly patients (*p* = 0.02), patients with tumour recurrences (*p* = 0.028), and patients with Grade 3 (*p* = 1.2e-08). We then estimated tumour microenvironment (TME) components in C1 and C2 and found that ImmuneScore, StromalScore, and ESTIMATEScore are higher, while tumour purity is worse in C1 (Figure 5A). The increase in these scores indicates an increase in the proportion of corresponding components in TME. The bulk gene expression patterns were examined using the CIBERSORT algorithmic technique, which allowed the percentages of 22 subgroups of tumour-infiltrating immune cells in various subtypes to be calculated (Figure 5B ). While the CD8^+^ T-cells, macrophages, and resting mast cells are more prevalent in the C1 subtype, the activated mast cells and eosinophils are more prevalent in the C2 subtype (Figure 5C). Meanwhile, Figure 5D shows all the statistically distinct immune checkpoint genes, which are all expressed at lower levels in C2. Our previous study found that low expression levels of these immune checkpoints genes were linked to a better survival probability in LGG patients (Wang et al., 2022). In addition, there were significant variations in immunotherapy responsiveness between C1 and C2 subtypes (Figure 5E). The C1 subtype had a lower exclusion score, illustrating that patients have a greater likelihood of gaining benefits from ICB (Figure 5F).

### Determination and verification of a cuproptosis and necroptosis-related genes-based prognostic signature

**FIGURE 6:**
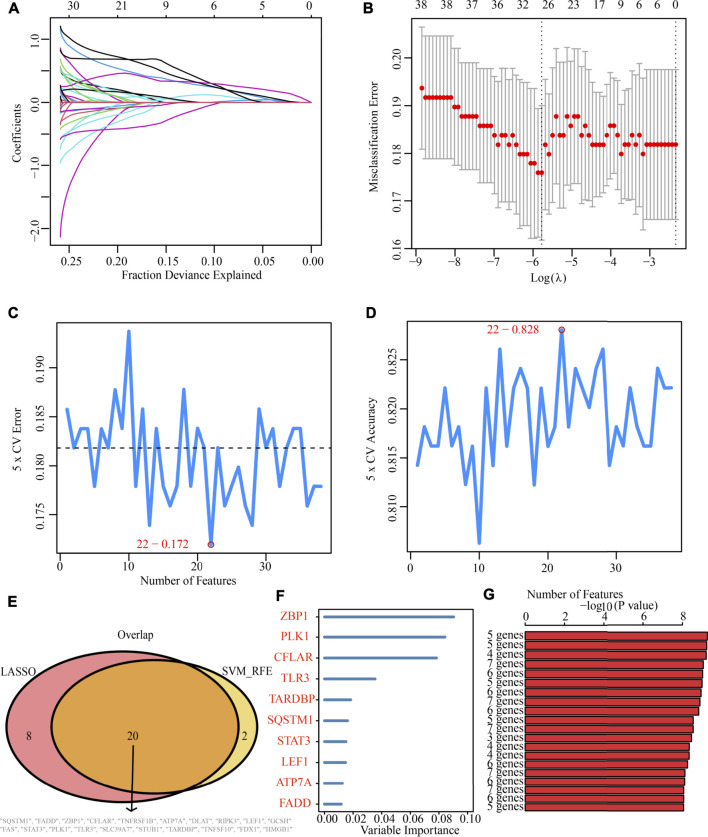
Machine learning identification of the optimal prognostic signature. **(A,B)** Identification of 28 CNRGs through the LASSO algorithm. **(C,D)** Identification of 22 CNRGs through the SVM-RFE algorithm. **(E)** Acquisition of 20 candidate CNRGs after intersecting LASSO and SVM-RFE algorithms. **(F,G)** Construction of a five-CNRG signature through random survival forests-variable hunting (RSFVH) algorithm.

**FIGURE 7:**
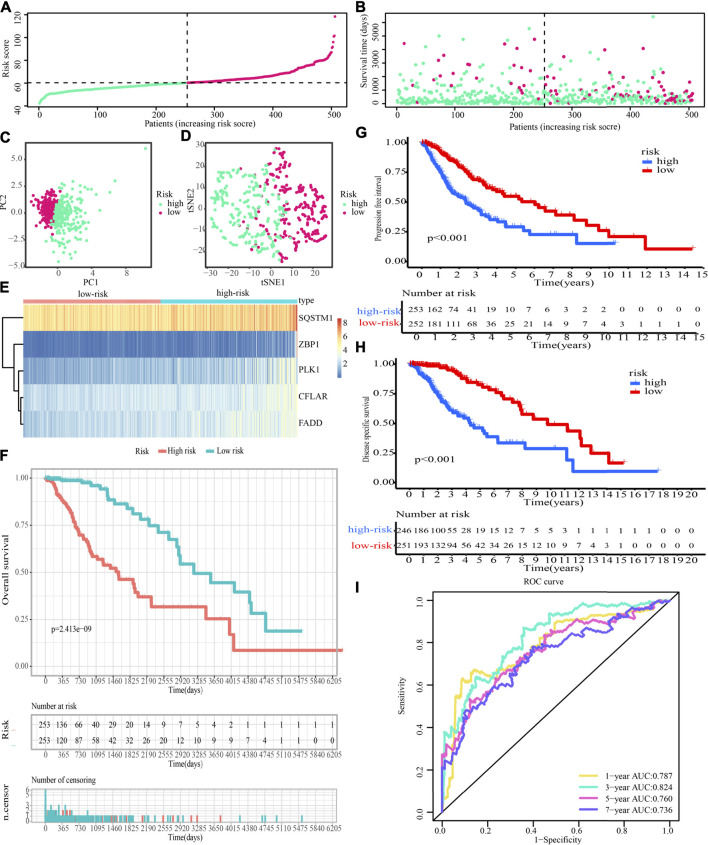
Evaluation of the prognostic significance of risk score in the training cohort. **(A,B)** Distribution of risk scores, patient survival time, and glioma status (The line with black dots represents the optimum threshold value for categorising patients into low- and high-risk populations). **(C,D)** PCA and t-SNE analysis illustrated an excellent clustering performance of the five-gene-based risk score. **(E)** The expression patterns of five CNRGs that were included in the signature as mapped out in a heatmap using the training dataset. **(F–H)** Survival curve of training cohort. **(I)** ROC curves of training cohort.

Additionally, after obtaining 38 prognostic CNRGs, we employed the LASSO technique to get a set of 28 CNRGs (Figures 6A,B), and the SVM-RFE algorithm to choose a set of 22 CNRGs (Figures 6C,D). Twenty potential CNRGs were identified after the intersection of the CNRGs that had been selected by the LASSO and SVM-RFE algorithms, and these CNRGs were then subjected to the RSFVH algorithm to further filter the genes. Following that, a novel CNRGs-based signature is established, risk score = 5.68460388028422 * ZBP1 + 5.58839133632066 * PLK1 + 6.382784047 * CFLAR + 3.560828639 * SQSTM1 + 3.541878806 * FADD (Figures 6E–G). Samples in the training cohort were categorised into low- and high-risk populations (Figure 7A). Figure 7B demonstrates that the group with a low risk exhibited a mortality rate that was lower in contrast with the group with a high risk. In both the PCA and the t-SNE analyses, the two risk groups hardly intersected, implying that it would be feasible to use the signature described above (Figures 7C,D). The heatmap shows the expression levels of the five CNRGs in our signature (Figure 7E). The high-risk patients reported worse OS, PFI, and DSS, as illustrated by the survival analysis (all *p* < 0.001) (Figures 7F–H). In addition, the time-dependent ROC curve analysis was done so that an accurate assessment of the signature could be made. The AUC values are 0.787, 0.824, 0.760, and 0.736 for 1-, 3-, 5-, and 7-year survival (Figure 7I). Meanwhile, in contrast with three other widely used prognostic signatures, our signature exhibited a much higher likelihood of correctly predicting patient survival (Supplementary Figure S7).

In addition, we analysed the potential relationship between our signature and cuproptosis, necroptosis, and cancer-related pathways. The findings highlighted that the CNGR signature was intimately linked to the cancer-associated pathways, as well as to cuproptosis and necroptosis (Supplementary Figure S8).

### Clinical characteristics, immune characteristics, and tumour stem features in low- and high-risk populations

**FIGURE 8:**
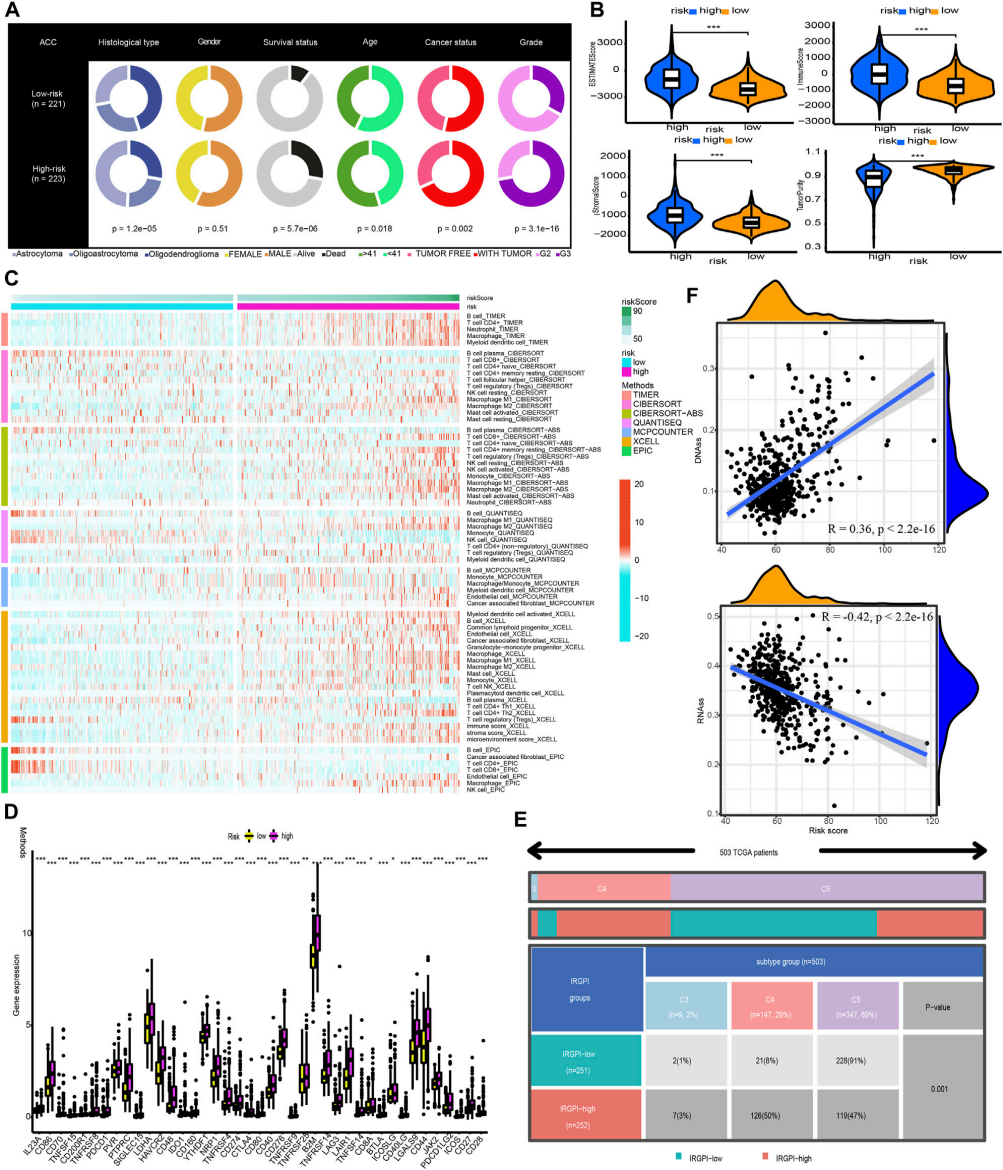
Clinical characteristics, immune characteristics, and tumour stem characteristics in the training cohort. **(A)** Pie charts illustrating the Chi-squared test of clinical and pathologic features between low- and high-risk categories **(B)** Analyses the similarities and differences between low-risk and high-risk groups in terms of TME components. **(C)** The immune cell infiltration landscape within the training cohort. **(D)** The expression profiles of ICGs in the training cohort. **(E)** Heat map and table illustrating the immune subtypes (C3, C4, and C5) distribution between low- and high-risk categories. **(F)** The correlation analysis between tumour stemness index and risk score.

Figure 8A shows that the high-risk population had a larger percentage of astrocytoma samples, whereas the low-risk population exhibited a greater percentage of oligodendroglioma samples. In the high-risk grouping, there are furthermore more deceased patients, elderly patients (>41 years old), patients with tumour recurrence, and G3 patients (all *p* < 0.05).

Afterwards, the tumour purity, ESTIMATE, immune, and stromal scores were computed by utilizing the ESTIMATE method to investigate the link between these factors and the CNRG scores. In comparison to the low-risk category, the high-risk category had a higher stromal, immune, and higher ESTIMATE score; nevertheless, it had a lower tumour purity (Figure 8B).

The variations in the immune cell components between the low- and high-risk groups were analysed and compared to get a deeper comprehension of the inherent association that exists between the risk score and the immunological environment of the LGG samples. Figure 8C is a heatmap that was generated using seven different algorithms, and it depicts the various immune cell components. Based on the TIMER, MCPCOUNTER, and XCELL algorithms, the proportion of B cells was elevated in the high-risk population, whereas the proportion of plasma cells decreased premised on the XCELL, CIBERSORT-ABS, and CIBERSORT algorithms. According to TIMER, the proportion of CD4^+^ T-cells in the population at low risk is lower. In the low-risk category based on CIBERSORT and CIBERSORT-ABS, naive CD4^+^ T-cells are more prevalent, while resting memory CD4^+^ T-cells are less prevalent. According to XCELL, the low-risk subgroup had a lower proportion of T helper 1 (Th1) and T helper 2 (Th2) cells. As per the CIBERSORT and CIBERSORT-ABS, the population at high risk has a greater abundance of CD8^+^ T-cells. According to CIBERSORT, CIBERSORT-ABS, and XCELL, the abundance of NK cells that fall into the high-risk group is much greater. As per the CIBERSORT-ABS, MCPCOUNTER, and XCELL, the abundance of monocytes that belong to the high-risk category is much greater. According to TIMER and MCPCOUNTER, the fraction of macrophages in the low-risk population is lower. Premised on the TIMER, MCPCOUNTER, and XCELL, a larger proportion of myeloid dendritic cells and neutrophil cells are seen in the high-risk category. These findings are also supported by TIMER and CIBERSORT-ABS. Additionally, based on CIBERSORT, CIBERSORT-ABS, QUANTISEQ, and XCELL, the high-risk group has a greater percentage of M1 and M2 macrophages in contrast with the low-risk population.

A weak local immune response could lead to increased immune cell infiltration as a coping mechanism. In high-risk LGG populations, ICG expression was increased (all *p* < 0.05, Wilcox test) (Figure 8D). The attenuation of effective anti-cancer immune responses caused by higher ICG expression led to immunocytes migrating into the TME to improve the compensatory response. In addition, we found that all LGG patients in TCGA cohort were associated with only C3, C4, and C5 immune subtypes (Figure 8E). The low-risk LGG population recorded a higher percentage of C5 immune subtypes in contrast with the high-risk population and a lower percentage of C3 and C4 immune subtypes (*p* = 0.001).

The tumour stem cell score not only reflects the pattern of intra-tumour heterogeneity but also correlates with immune infiltration and immunological checkpoints. We can better comprehend the TIME and create new targeting medications for ICB therapy by thoroughly analysing tumour stem cell scores. We then examined the correlation between our CNRG signature and tumour stemness index. The results illustrated that the risk score had a positive connection with DNAss (*R* = 0.36, p 2.2e16) and an inverse link to RNAss (*R* = −0.42, p 2.2e16) (Figure 8F).

### Tumour mutation profile and immunotherapy response prediction in high- and low-risk populations

**FIGURE 9:**
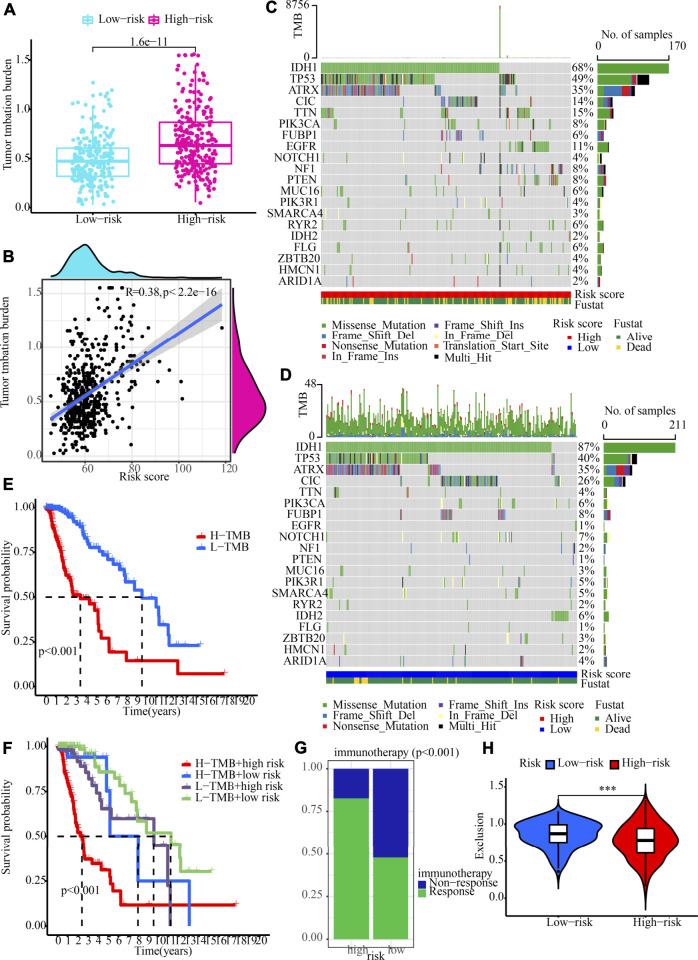
TMB analysis and immunotherapy response prediction in the training cohort. **(A)** The variation between high- and low-risk groups in terms of TMB. **(B)** An analysis of the correlation between risk score and tumour mutation burden **(C,D)** OncoPrint of frequently mutated genes in high- and low-risk groups. **(E)** The Kaplan-Meier curve of overall survival for patients, as shown by samples categorised according to their TMB score. **(F)** OS for patients defined by the samples categorised by both their risk score and their TMB score, as shown by the Kaplan-Meier curve. **(G)** The variation in immunotherapy response in low- and high-risk groups. **(H)** Immunotherapy response prediction in the training cohort.

We determined the value of the TMB by comparing the two risk populations, taking into consideration the strong link between the TMB and the effectiveness of immunotherapy. TMB quantification showed that the high-risk category recorded a greater TMB, which was in line with our expectations (*p* = 1.6e-11; Figure 9A). Additionally, Spearman correlation analysis illustrated a moderately positive link between risk score and TMB. (*R* = 0.38, *p* < 2.2e-16; Figure 9B). We also evaluated the variations in LGG driver genes between low- and high-risk groupings. Figures 9C,D displays driver genes with a high change frequency, such as IDH1, TP53, ATRX, CIC, and TTN. In addition, IDH1 and CIC mutation frequencies were greater in the low-risk category, while TP53 and TTN mutation frequencies were greater in the high-risk category. Patients who had a low TMB gained a satisfactory survival benefit (Figure 9E). After that, we examined whether or not it would be beneficial to use TMB in conjunction with the risk score to anticipate patients’ outcomes. As per the findings of the KM analysis, a lower risk score and a lower TMB are associated with a greater likelihood of surviving (Figure 9F). Moreover, when evaluating the efficacy of immunotherapy, we focused our attention primarily on determining the significance of the risk scores. The findings demonstrated that the relative odds of responding favourably to immunotherapy in the high-risk category were much greater in contrast with those in the low-risk category (Figure 9G). A lower exclusion score was associated with high-risk LGG populations, indicating that these LGGs populations would be less likely to evade immunotherapy (Figure 9H). All of these data illustrate that patients in the high-risk category would gain more benefits from ICB treatment. Therefore, we propose that our signature can be applied to clinical patients to accurately predict whether or not they would benefit from immunotherapeutic interventions.

### Independent prognostic performance of our cuproptosis and necroptosis-related gene signature and nomogram plot establishment

**FIGURE 10:**
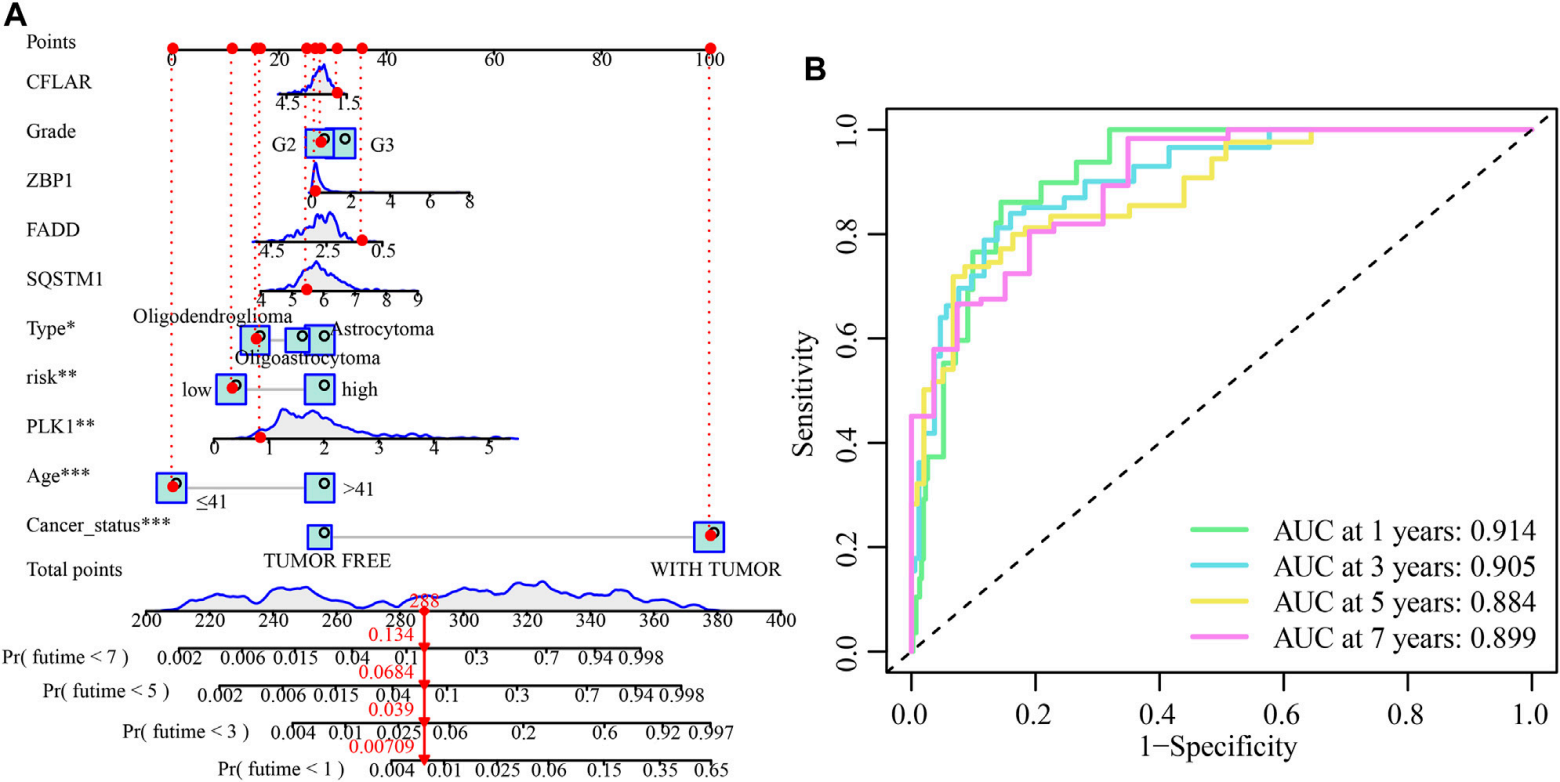
The development and validation of the risk score-based nomogram. **(A)** A nomogram of LGG was used to predict 1-year, 3-year, 5-year, and 7-year survival rates. **(B)** The AUC values of the ROC curves for improved evaluation of the prognostic ability of the nomogram.

Both univariate and multivariate Cox regression analyses were carried out to evaluate whether the CNRG score is independent of other clinical variables such as tumour type, gender, age, cancer status, and grade (Table 1). The results suggested that age, cancer status, grade, and CNRG score independently functioned as prognostic indicators. Although tumour type did not independently act as a prognostic indicator (*p* = 0.061), we consider this factor to be non-negligible. Following that, we devised a nomogram for OS prediction by making use of clinical data and risk scores in TCGA dataset (Figure 10A). The predictors of the nomogram consisted of the above independent prognostic indicators, tumour type, and five model genes. The AUC values of the ROC curves were 0.914, 0.905, 0.884, and 0.899, correspondingly, indicating the nomogram had excellent prognostic performance (Figure 10B).

**TABLE 1:** Univariate and multivariate Cox regression analysis determined the independent prognostic performance of our risk score.

Univariate	HR	HR.95L	HR.95H	*p* value	Multivariate	HR	HR.95L	HR.95H	*p* value
^a^ **Type**	0.720494516	0.561649	0.924265	0.009888	^a^ **Type**	0.777666	0.597662	1.011884	0.061204
^b^Gender	0.912138	0.594411	1.399697	0.673813	^b^Gender	1.12804	0.724577	1.756162	0.59371
^c^ **Age**	4.376072	2.722401	7.034235	1.09E-09	^c^ **Age**	3.47046	2.061005	5.843794	2.87E-06
^d^ **Cancer_status**	39.2837	5.466809	282.2869	0.000264	^d^ **Cancer_status**	33.08822	4.589435	238.5545	0.000517
^e^ **Grade**	3.694602	2.280463	5.985662	1.10E-07	^e^ **Grade**	2.007173	1.136654	3.54439	0.01633
^f^ **RiskScore**	1.062957	1.048184	1.077939	1.23E-17	^f^ **RiskScore**	1.036923	1.02053	1.053579	8.21E-06

a Type: Astrocytoma, Oligoastrocytoma, Oligodendroglioma.

b Gender: Female, Male.

c Age: ≤41, >41.

d Cancer_status: Tumor free, With tumor.

e Grade: G2, G3.

f RiskScore: risk score = 5.68460388028422 * ZBP1 + 5.58839133632066 * PLK1 + 6.382784047 * CFLAR + 3.560828639 * SQSTM1 + 3.541878806 * FADD.

### Prognostic significance of the cuproptosis and necroptosis-related gene signature in the validation set

**FIGURE 11:**
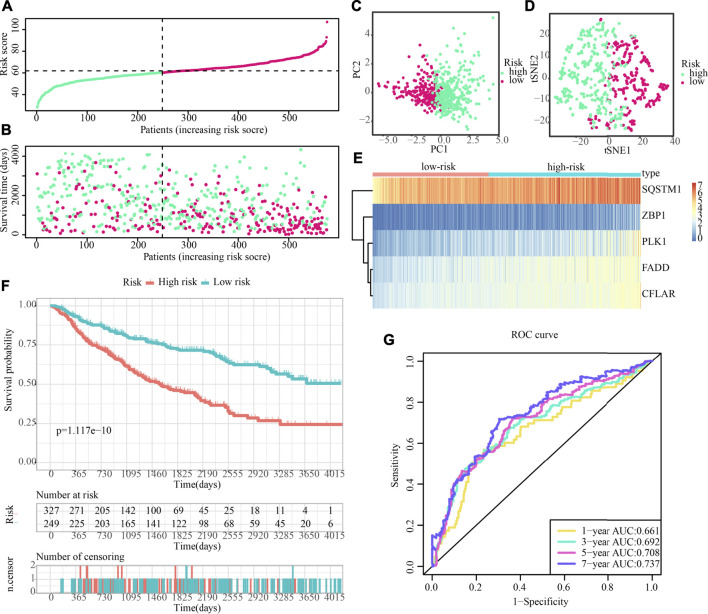
The predictive performance of the risk score was validated by the validation cohort. **(A)** Group division in the validation cohort. **(B)** High-risk patients exhibited an increased incidence of death. **(C,D)** PCA and t-SNE analysis demonstrated an excellent clustering performance of the five-gene-based risk score. **(E)** Heatmap of the expression profiles of five CNRGs included in the signature in the validation cohort. **(F)** Survival curve in CGGA cohort. **(G)** ROC curves in CGGA cohort.

The predictive accuracy of the 5-CNRGs prognostic signature was confirmed in the CGGA cohort to figure out whether or not it had the same prognostic significance across a variety of groups. The samples from the LGG were categorized into two groups using the same threshold values as that used for the samples from the TCGA cohort (Figure 11A). Patients who have high-risk scores have a shortened survival time as well as an increased likelihood of death (Figure 11B). In both the PCA and the t-SNE analyses, the two risk groups hardly overlapped, implying that it would be feasible to use the signature described above (Figures 11C,D). The heatmap demonstrates that the levels of the five CNRGs expressions in our signature agree with the values in the calculation equation (Figure 11E). The KM survival curves demonstrated a statistically significant difference in OS between the low- and high-risk groups (Figure 11F). The AUC values were 0.661, 0.692, 0.708, and 0.737 over 1, 3, 5, and 7 years, demonstrating that the model has a considerable predictive ability (Figure 11G).

**FIGURE 12:**
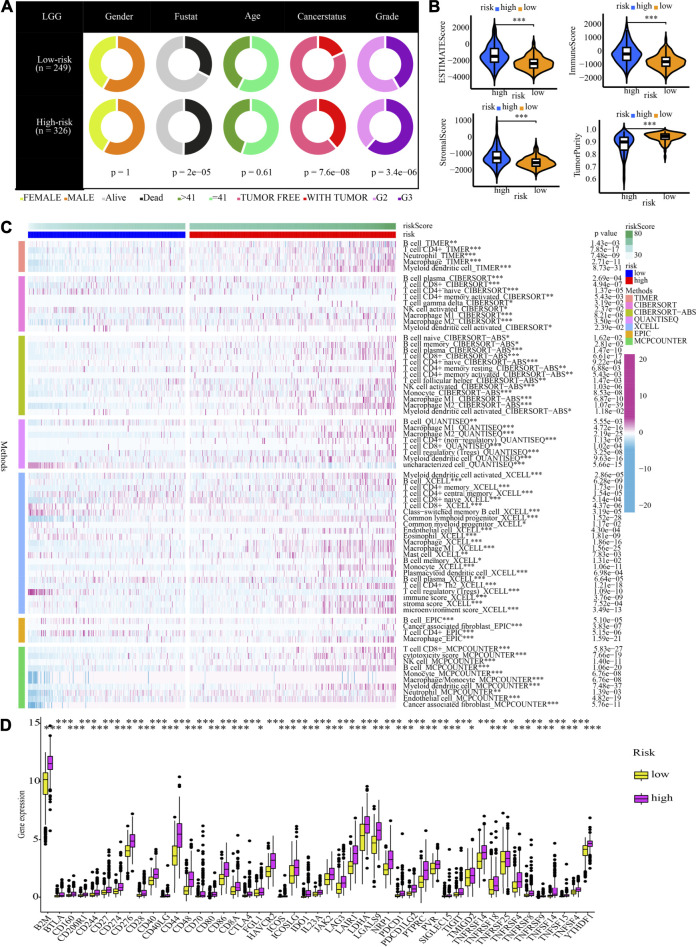
Clinical characteristics and immune characteristics in the validation cohort. **(A)** Analysis of the compositional variations between low- and high-risk groups in terms of clinical characteristics. **(B)** Comparative analysis of the TME components for low- and high-risk groups **(C)** The pattern of the distribution of immune cell infiltration in the validation cohort. **(D)** The expression profiles of ICGs in the validation cohort.

The differences in clinical traits across populations at high- and low-risk were then depicted in Figure 12A. The high-risk demographics had more G3 patients, more dead people, and more tumour recurrences. Similar to this, high-risk populations in the CGGA cohort had higher ESTIMATEScore, ImmuneScore, and StromalScore values and lower tumour purity values (Figure 12B). Figure 12C also showed the significant infiltration levels of immunocytes in the populations at low and high risk. The infiltration levels of myeloid dendritic cells, CD4^+^ T-cells, Th2 cells, macrophages, and B cells were increased in the high-risk category. Meanwhile, the contrast in ICG expression between populations at high- and low-risk illustrated the same patterns. High-risk groups showed higher ICG expression compared to low-risk populations, which may be the cause of a likely compensating rise in immune cell infiltration (Figure 12D).

### Immunohistochemistry and immunofluorescence of five cuproptosis and necroptosis-related genes in low-grade glioma tissues

**FIGURE 13:**
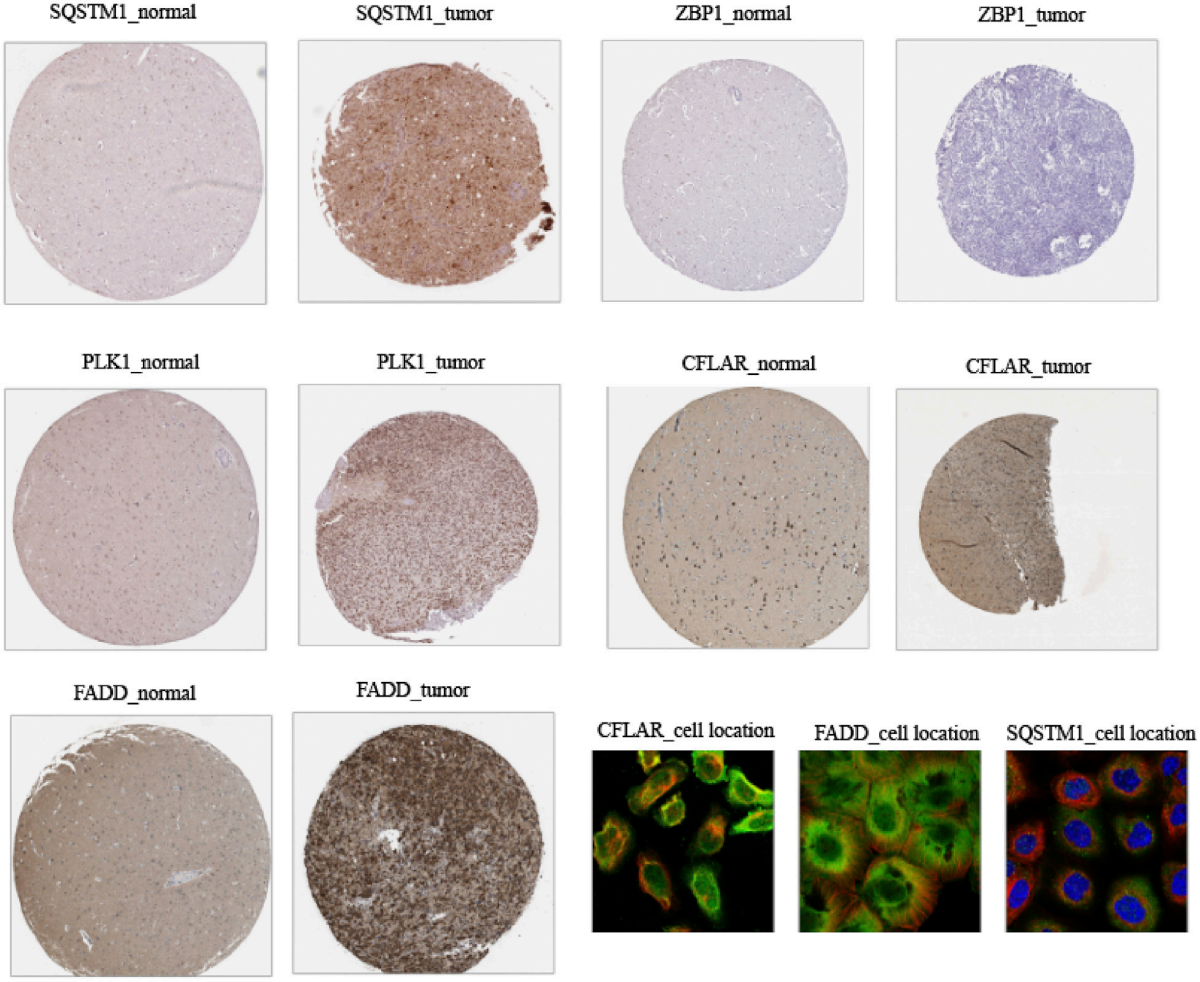
Immunohistochemistry and immunofluorescence of clinical samples (tumour tissues vs. normal adjacent tissue).

The IHC staining images for the model gene-related proteins in LGG and normal lung tissues were retrieved from the HPA database and used in determining whether or not these five CNRGs exhibit differentially high levels of protein expression in LGG. In line with the findings described above, the analysis revealed that the levels of protein expression for CFLAR, FADD, PLK1, and SQSTM1 in LGG samples were remarkably elevated in contrast with those in normal samples (Figure 13). We then explored the cellular localization of these genes, of which ZBP1 and PLK1 were not found. The expression product of CFLAR, FADD, and SQSTM1 were mainly located on the Vesicles and Cytosol, Nucleoplasm, Plasma membrane, Cytosol and Nuclear bodies, Nucleoplasm and Cytosol respectively (Figure 13).

## Discussion

It has been determined that LGG is a class of primary brain tumours that develops from supporting glial cells. In-depth mechanisms of LGG are supposed to be heavily researched due to its uncertain pathophysiology and unsatisfactory treatment results. Cuproptosis and necroptosis, two new forms of cell death, might be able to provide a fresh approach to the therapy of malignancies. We started by examining the variations in the expression of 87 CNRGs and evaluating whether or not these genes served as protective or risk factors in various cancers. We found that the majority of CNRGs functioned as risk genes in LGG patients. Furthermore, apparent CNV and SNV alterations of CNRGs were also found in LGG populations, suggesting the crucial role of CNRGs in LGG. Cancer signalling analysis of CNRGs found most oncogenic pathways were significantly enriched in LGG. To anticipate the clinical outcomes and immunotherapy response of LGG patients based on CNRGs, we first categorised molecular subtypes and then created and verified a unique multigene signature.

38 CNRGs with prognostic values were found for NMF clustering and signature building. First, CNRGs are applied to divide LGG samples into two molecular clusters with significantly distinct prognoses, clinical traits, and immune microenvironment. ImmuneScore, StromalScore, and ESTIMATEScore were generated to infer the stromal and immunological components of each patient. TME is a niche consisting of cytokines, chemokines, and stromal cells that sustain tumour tissue (Belli et al., 2018). Higher ImmuneScore and StromalScore values are related to larger, respective TME components. The findings imply that C1 subtypes with a worse prognosis may have a more abundant immune abundance. Elevated infiltration levels of CD8^+^ T-cells, macrophages, and resting mast cells were found in C1 subtypes after further analysis of 22 immune cell infiltration components in each LGG sample using the CIBERSOFT algorithm. Targeting the remodelling of the TME might be a viable treatment method that could attenuate the growth of tumours. Numerous research reports have shown that the immune microenvironment affects the biological activity of tumours (Mlecnik et al., 2016; Angelova et al., 2018; Mao et al., 2021). Furthermore, we discovered that immune checkpoint genes are expressed at a high level in the C1 subtype and are linked to an unfavourable survival prognosis. Collectively, the high infiltration levels of immune cells in the C1 subtype may be a local compensatory phenomenon of active immune checkpoints. Despite the presence of high anti-cancer immunity in TME, ICG expression is also higher in the C1 subtype, which suppresses the immune cell functions and prevents the body from generating an effective anti-tumour immune response, leading to tumour immune evasion and resulting in a poor prognosis for the high-risk subgroup of patients Our data point to the possibility that patients with a C1 subtype might benefit more from immunotherapy. We further verified our conjecture by using the TIDE database. Significant statistical difference was observed for TIDE-derived immunotherapy response prediction and exclusion score between the two subtypes. The exclusion score is a negative biomarker of immunotherapy and its downregulation provided an essential grounding for immunotherapy response prediction (Fu et al., 2020). Our results found that the C1 subtype is characterized by a lower exclusion score and showed a higher proportion of immunotherapy response, suggesting that immunotherapy is more reliable and applicable to the C1 population.

Following that, a novel CNRG signature involving SQSTM1, ZBP1, PLK1, CFLAR, and FADD was developed and validated to predict survival and benefit from immunotherapy. Importantly, our signature outperforms the other three signatures for predicting survival and has a good diagnostic value. SQSTM1 is a versatile stress-inducible scaffold protein responsible for regulating a wide range of cellular activities (Clausen et al., 2010), including nuclear factor kappa-B signalling. Additionally, it establishes a link between autophagy and polyubiquitinated cargo (Liu et al., 2017). In glioma, SQSTM1 could promote proliferation, invasion and mesenchymal transition (Polonen et al., 2019), which accurately predicted the prognosis of patients (Li et al., 2019b). Meanwhile, SQSTM1 is implicated in other numerous types of disorders, particularly, neurodegenerative (Ma et al., 2019), cardiometabolic disorders (Jeong et al., 2019), melanomas (Karras et al., 2019) and breast cancer (Ryoo et al., 2018). The role of ZBP1 in tumour progression and metastasis is unclear. Recently, research has illustrated that ZBP1 is highly increased in mice and humans with late-stage tumours and that ZBP1 deletion inhibits tumour metastasis in preclinical cancer models (Baik et al., 2021). Although the pivotal role of ZBP1 in LGG has not been reported before, ZBP1 expression was found to be significantly up-modulated in ovarian and colon cancer and linked to poor prognosis (Gu et al., 2004; Dimitriadis et al., 2007). PLK1 is closely associated with cell proliferation and has been intensively studied. PLK1 expression is dysregulated in several human cancers, including melanoma, breast, colorectal, gastric, and lung cancers (Strebhardt, 2010). It has been reported that PLK1 inhibits glioma cell invasiveness and induces apoptosis in glioma cells (Wang et al., 2020). CFLAR is a known key regulator of the apoptotic signalling pathway and is abnormally expressed in a variety of cancers. Besides regulating apoptosis at different levels of the signalling cascade, there is growing evidence that CFLAR is also involved in the control of alternative cell death pathways, for example, necroptosis and autophagic cell death (Fulda, 2013). CFLAR is also considered a promising therapeutic target, and multiple approaches have been developed to interfere with CFLAR expression or function in human cancers (Fulda et al., 2000; Panner et al., 2005; Haag et al., 2011). FADD is a key bridging protein that mediates apoptotic signalling (Mouasni and Tourneur, 2018). FADD is not only linked to apoptosis but also proliferation, innate immunity, tumour growth, inflammation, and autophagy (Schwarzer et al., 2020). Thus, FADD is an important and specific controller in many important cellular processes (Tourneur and Chiocchia, 2010). At the same time, FADD overexpression inhibits proliferation while promoting apoptosis in human GBM cells (Wang et al., 2017).

It is widely known that TIME is intimately linked to carcinogenesis and cancer development (Petitprez et al., 2020; Chen et al., 2021). Immune cells may act in a tumour-promoting or tumour-antagonistic manner. Although tumour-antagonising immune cells within the TME tend to target and destroy cancerous cells in the initial phases of oncogenesis, tumour cells appear to eventually evade immune surveillance and even block the cytotoxic function of tumour-antagonising immune cells *via* a variety of processes (Lei et al., 2020). In subsequent explorations of the TME, we found that several cancer-promoting immune cells, such as Th2 (Bing et al., 2017; Watt et al., 2017; Yu et al., 2022), and M2 macrophage (Mantovani et al., 2002; Zhu et al., 2020), are up-modulated in the high-risk category, although some anti-tumour immune cells had higher proportions, such as B cell (Sarvaria et al., 2017), M1 macrophage (Najafi et al., 2019), NK cells (Terren et al., 2019), and mDC (Banchereau et al., 2009; Lebre and Tak, 2009). Plasma cells exhibited lower proportions as a result of substantial intake in the high-risk category to carry out their anti-tumour activity. As a consequence of antigen exposure, naïve CD4^+^ T-cells concurrently undergo the process of transformation into memory T-cells (Obst et al., 2005). The high-risk category demonstrated a decline in naive CD4^+^ T-cells and an elevation in T-cell memory. Moreover, cancer cells may trigger many immunological checkpoint pathways with immunosuppressive properties (Darvin et al., 2018). Therefore, these cancer-promoting immune cells and ICGs are expected to be potentially effective therapeutic targets.

TMB may be used as an indicator to predict ICB effectiveness and has become a biomarker in some cancer types to identify individuals who would benefit from immunotherapy, according to reports (Chan et al., 2019; Tian et al., 2022). At the same time, we found that the proportion of TMB in the high-risk category is higher. IDH1, TP53 and ATRX are the leading three genes exhibiting the greatest mutation frequency in LGG. IDH1 mutations have been shown to improve LGG prognosis and lower-grade gliomas that had mutations in IDH but did not have 1p/19q codeletion virtually always also had mutations in ATRX inactivation (86%) and TP53 (94%) (Cancer Genome Atlas Research et al., 2015). Additionally, in data spanning all WHO grades, changes in ATRX strongly correlated with mutations in TP53 (*p* < 0.0001) and IDH1/2 (*p* < 0.0001) (Liu et al., 2012). Higher frequencies of mutation in the high-risk cohort for IDH, TP53, and ATRX suggest a possible relationship between the three genes, and the combination of these three changes may result in the discovery of a new therapeutic target.

Finally, we discovered that the proportion of patients who responded favourably to immunotherapy was greater in the high-risk category, but the capacity of immune cells to evade immune surveillance was lower. All of these data illustrate that patients in the high-risk segment might gain more benefits from ICB. Therefore, We propose that our signature may be applied to clinical patients to accurately predict whether or not they would respond to immunotherapy.

In addition, we discovered that age, cancer status, grade, and risk score could independently function as prognostic indicators. We constructed a nomogram with predictors including tumour type, age, cancer status, grade, risk score, and five model genes. The AUC values of the ROC curves of the nomogram were satisfactory, which indicates the strong predictive power of the nomogram. Eventually, we verified the differential expression of related genes encoding proteins in the model using IHC data from the HPA database.

Our research has several drawbacks as well. Firstly, we only verified the CNRG-based signature using retrospective data from the CGGA and TCGA databases; in the future, we should examine its therapeutic significance by conducting more prospective investigations. Secondly, we need more large prospective clinical studies to assess its effectiveness and applicability. Thirdly, cuproptosis and necroptosis need to be investigated extensively in both *in vivo* and *in vitro* settings before their potential roles in the onset and progression of LGG can be fully comprehended.

In summary, we developed the 5-CNRG-related signature to predict the prognosis and immunotherapy effectiveness among LGG patients. This signature has been well-validated from different points of view.

## Data Availability

The datasets presented in this study can be found in online repositories. The names of the repository/repositories and accession number(s) can be found in the article/Supplementary Material.

## Supplementary material

SUPPLEMENTARY FIGURE S1 Spearman correlations between CNRGs in both TCGA and CGGA cohorts.

SUPPLEMENTARY FIGURE S2 Single nucleotide variation (SNV) in pan-cancer.

SUPPLEMENTARY FIGURE S3 Copy number variation (CNV) in diverse types of cancers.

SUPPLEMENTARY FIGURE S4 Enrichment analysis for cancer signalling.

SUPPLEMENTARY FIGURE S5 Rank survey of NMF clustering.

SUPPLEMENTARY FIGURE S6 Consensus map of NMF clustering.

SUPPLEMENTARY FIGURE S7 Comparative analysis. The C-indices of our, Zheng’s, Lin’s, and Zhang’s signatures. **(B,C)** ROC curves and survival analysis of our signature. **(D–F)** ROC curves of Lin’s, Zhang’s, and Zheng’s signatures. **(G–I)** Survival analysis of Lin’s, Zhang’s, and Zheng’s signatures.

SUPPLEMENTARY FIGURE S8 Enrichment analysis of the CNRG signature to examine the potential relationship of the CNRG signature with cuproptosis, necroptosis, and cancer signalling.
