# Supplementary figures and images for: Machine learning identification of cuproptosis and necroptosis-associated molecular subtypes to aid in prognosis assessment and immunotherapy response prediction in low-grade glioma
Source: Front Genet. 2022 Sep 12;13:951239. doi: 10.3389/fgene.2022.951239 (PMC9524234; doi:10.3389/fgene.2022.951239)

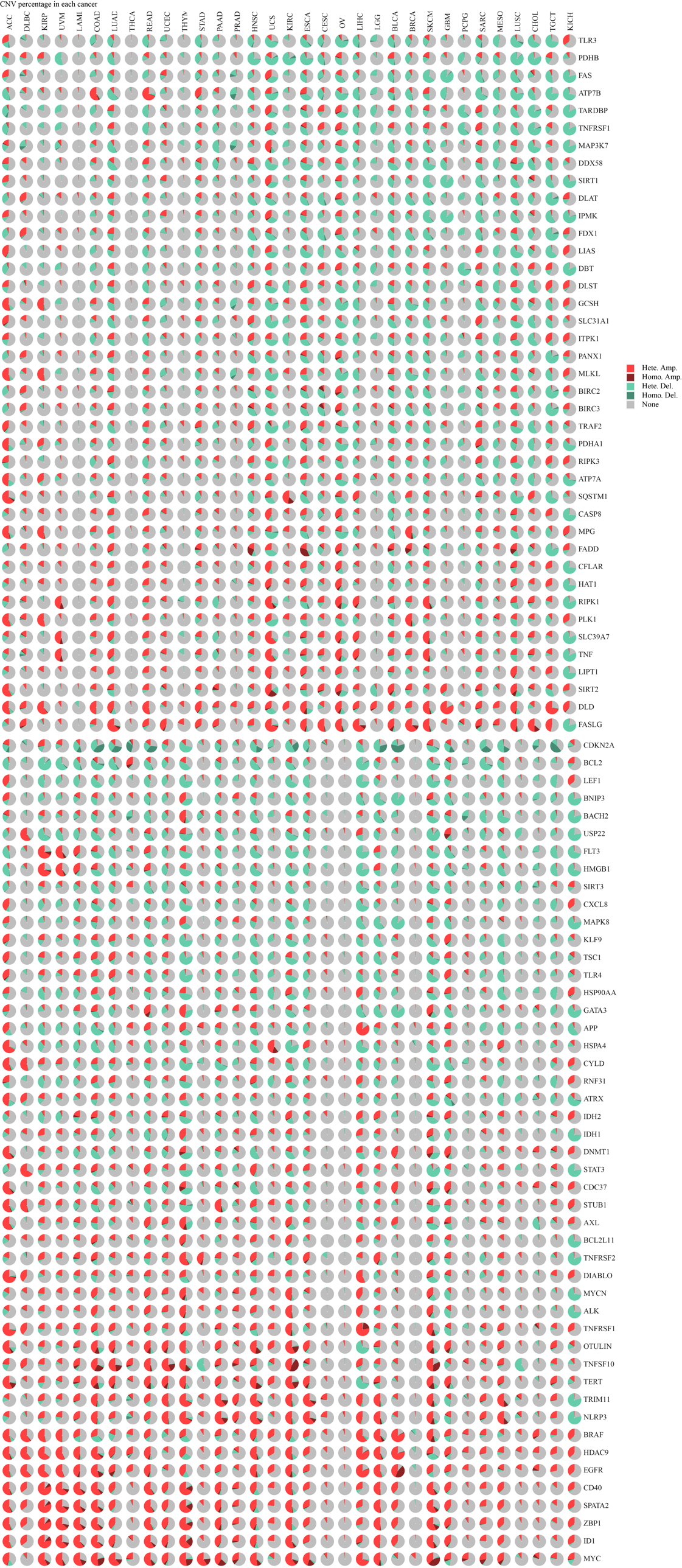

Supplement: Supplementary file 1 [file Image3.JPEG]

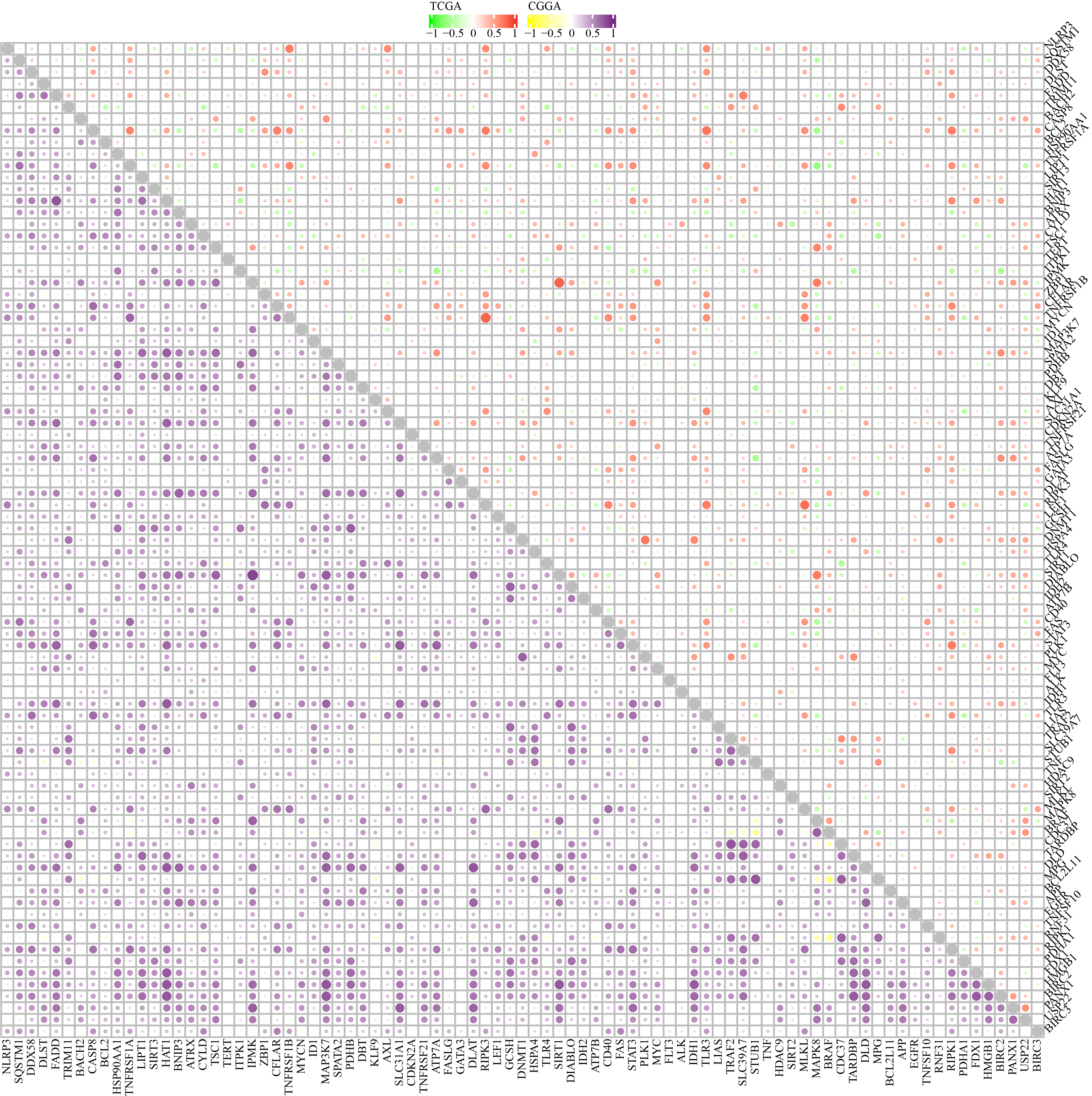

Supplement: Supplementary file 3 [file Image1.JPEG]

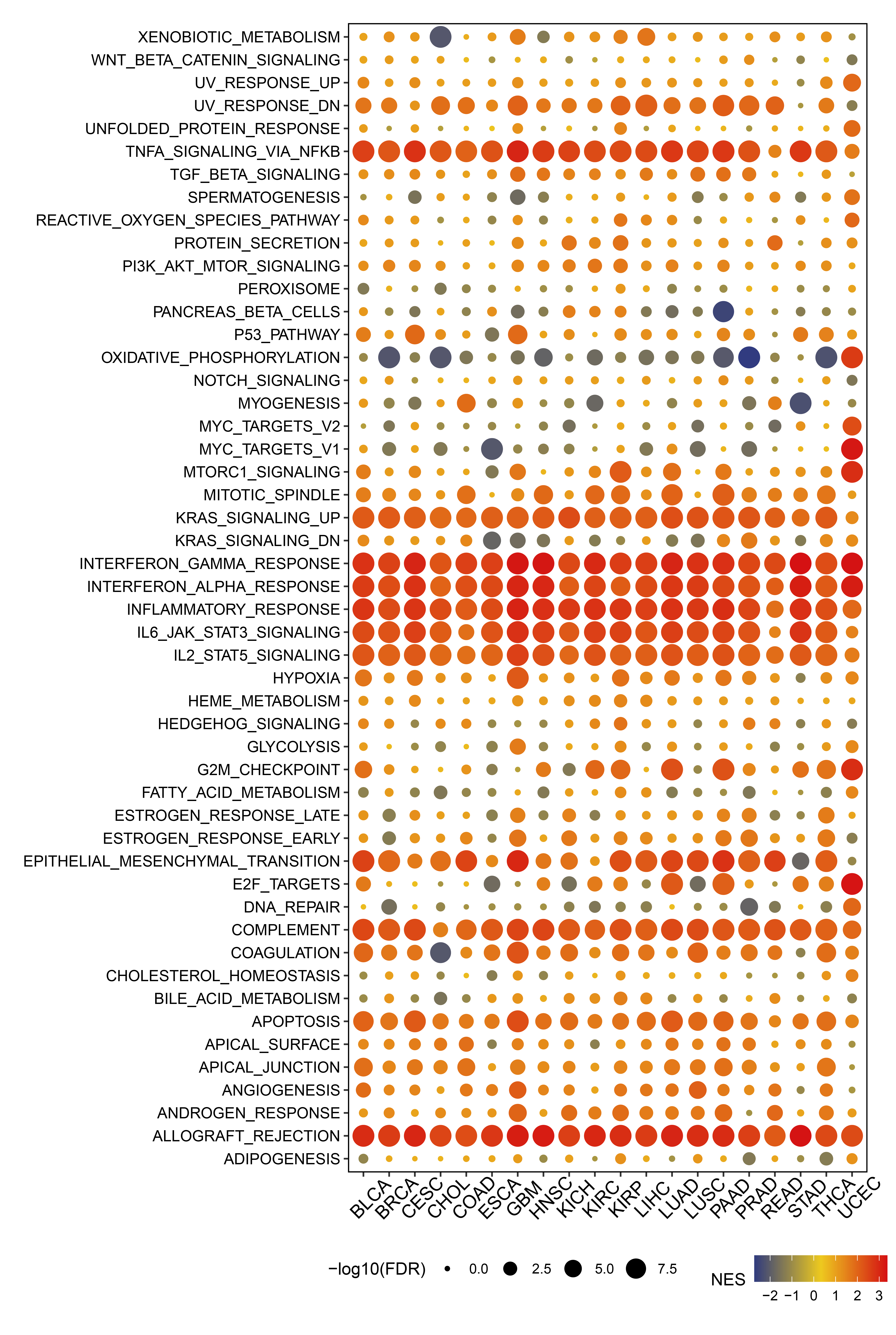

Supplement: Supplementary file 4 [file Image4.JPEG]

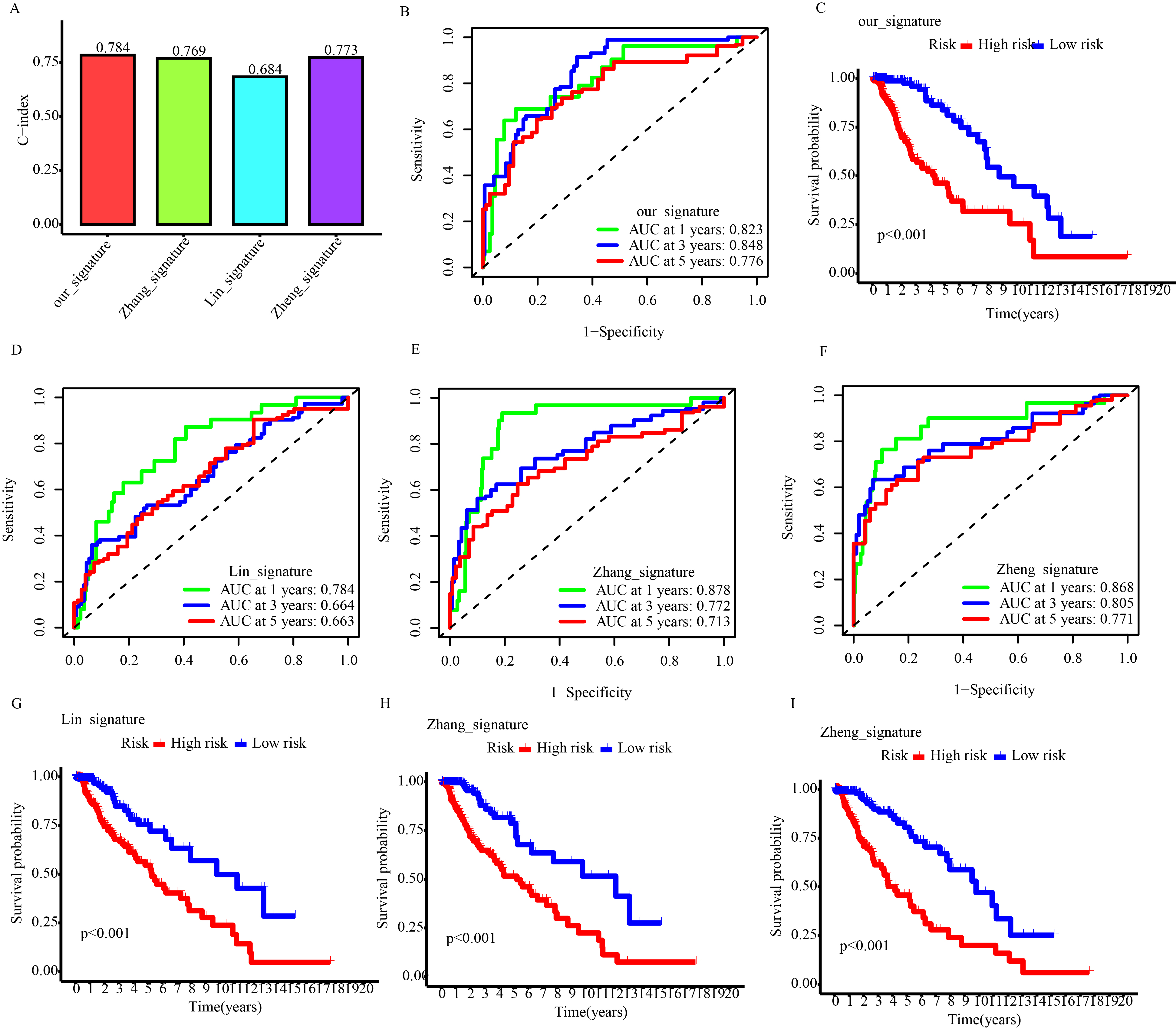

Supplement: Supplementary file 5 [file Image7.JPEG]

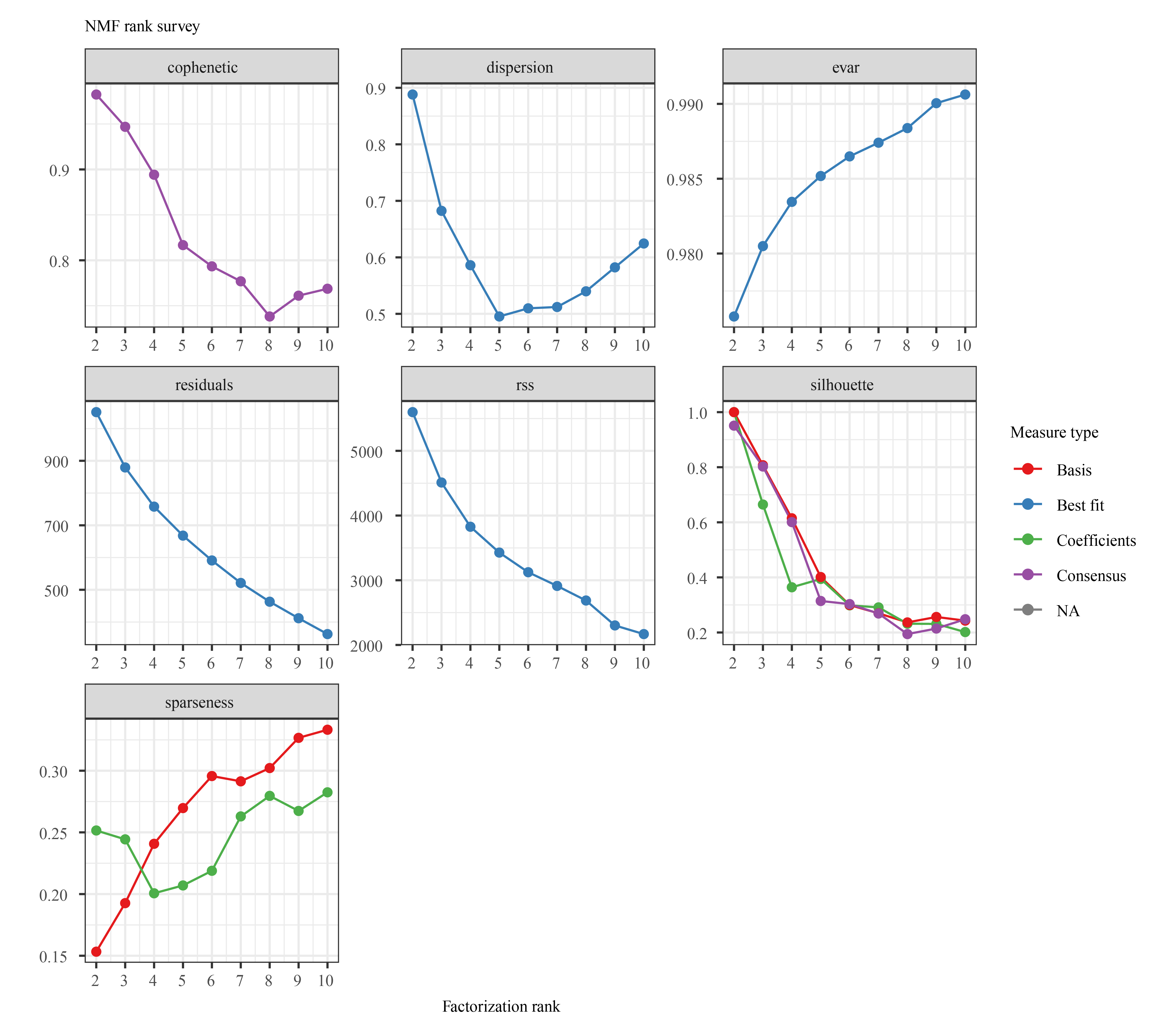

Supplement: Supplementary file 7 [file Image5.JPEG]

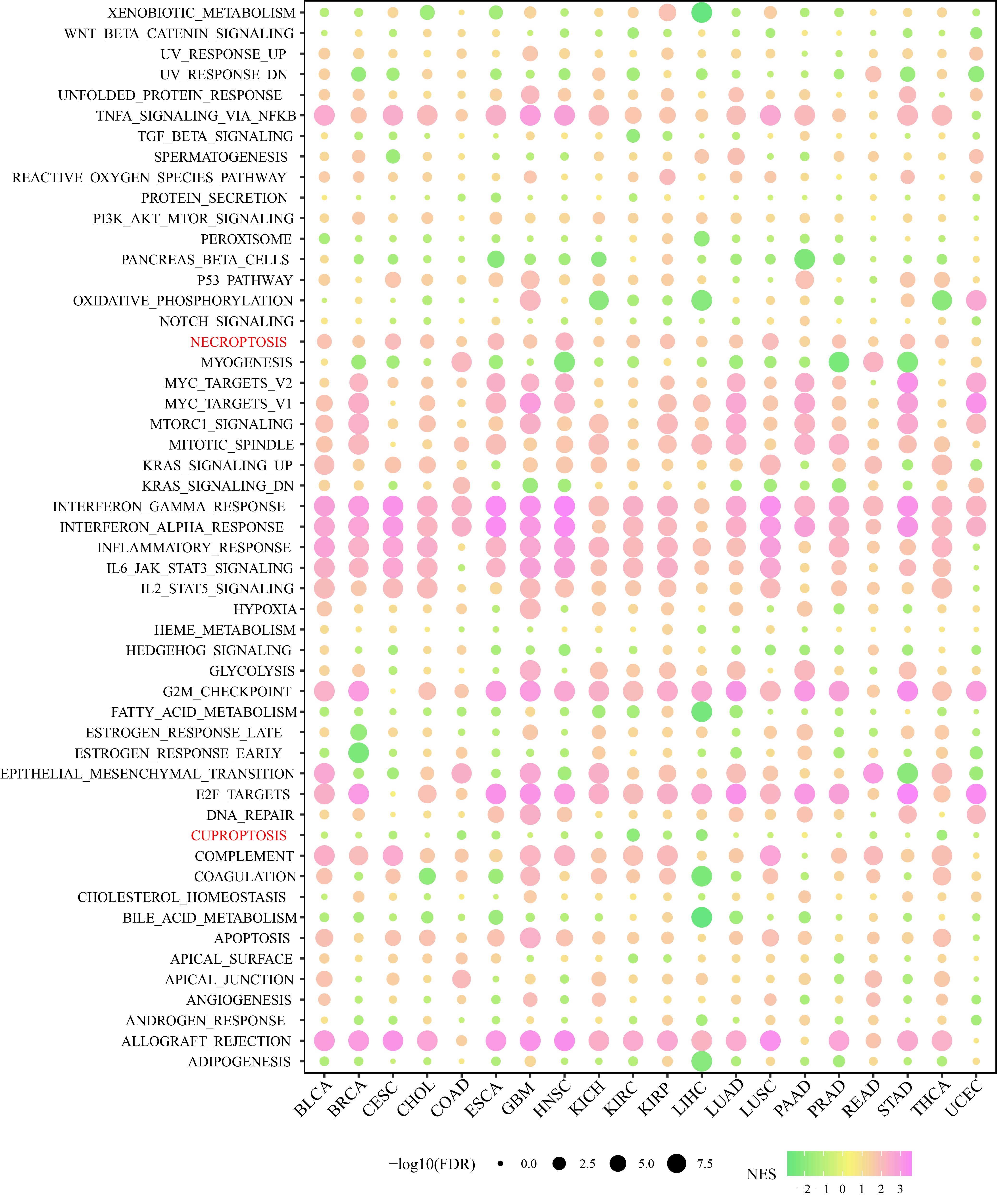

Supplement: Supplementary file 9 [file Image8.JPEG]
